# Case of Aneurism of the Popliteal Artery, in Which the Femoral Artery Was Tied with Success, by J. T. Simpson, Esq. With the Dissection of the Limb Nearly Two Years Afterwards, the Patient Having Died of Aneurism of the Aorta, Which Burst into the Pericardium

**Published:** 1827-06

**Authors:** Thomas Rose


*Case of Aneurism of the Popliteal Artery, in which the Femoral Artery was tied with success, by* J. T. Simpson, Esq. *With the Dissection of the Limb nearly two years afterwards, the Patient having died of Aneurism of the Aorta, which burst into the Pericardium.* Communicated by Thomas Rose, Esq.

Joseph Hancock, twenty-eight years of age, a soldier in the Coldstream Guards, by trade a brass-founder, was admitted into the regimental hospital, in the beginning of November, 1804, with an aneurism of the popliteal artery on the right side. About two months before, whilst he was in camp with the first battalion, he complained of pain and stiffness in his right ham, and, on examination, a small aneurismal tumor was then perceived. This gradually enlarged, and, at the time of his being received into the hospital, was of the size of a hen's egg. His knee was also stiff; his leg contracted, and slightly œdematous about the ankle. He did not recollect any cause to which he could refer the disease; but, when employed at his trade, he was in the habit of turning a large wheel with his right foot, which required considerable exertion.

On the 23d of November, the artery was cut down upon in the middle of the thigh, by Mr. Simpson, and secured by a single ligature. Every thing went on favourably, and on the 14th January he was dismissed from the hospital, the leg being supported by a flannel roller, which he was directed to wear for two or three months. In the beginning of April, no remains of the tumor could be perceived, the leg was in all respects as strong as the other, and he was sent to his duty.

From that time till the 13th August, 1806, he continued perfectly well, and, when not on regimental duty, employed himself as formerly at his trade. On that day, about five in the afternoon, whilst standing by the vice in the workshop, where he had been busy during the day, he was suddenly seized with faintness, and, leaning down his head on the vice, he observed to some of the workmen near him that he felt very ill. He had scarcely said so, when he dropt down, and, in less than five minutes, expired.

I had seen him about a week before his death, and had examined him particularly. His right leg was as muscular and strong as the other; and he told me that his health had never been better, and that he was as able to do his duty as in any former part of his life.

I examined the body on the 15th of August. The pericardium was found to contain about a pint of serum, and nearly an equal quantity of coagulated blood. The opening through which this had escaped was found in the anterior part of the aorta, about three-quarters of an inch beyond the semilunar valves. It was only large enough to admit the end of the smallest sized probe. On cutting into the heart and aorta, the left ventricle, particularly towards its apex, was found to be much thinner than natural, not appearing so strong as either of the auricles. Immediately beyond the valves, the internal coat of the aorta was much thickened, and thrown into rugæ, having a dark opaque colour, from effused blood between this and the muscular coat. In this diseased portion of the vessel, which was upwards of an inch and a half in length, two aneurismal pouches were formed,—one on the anterior convex side, large enough to contain three-quarters of an ounce of blood; the other directly opposite, and about half that size. On the internal surface of the larger sac, at its upper part, where it was most exposed to the impetus of the blood, a small round depression was observed, nearly two-tenths of an inch in diameter, having jagged edges, and being distinctly produced by ulceration. In the centre of this was the small opening which communicated with the pericardium. The same thickened and corrugated appearance was met with on the vessel, at the upper part of its arch, where the three great trunks are given off; and a similarly diseased spot, about the size of a shilling, was found in the aorta descendens, three inches below the arch. Ossification had not taken place to any extent in the aorta, but some opaque white specks were seen in different parts of its coats.

The other vessels, and the viscera of the thorax and abdomen, were free from disease.

The thigh was injected from the external iliac artery. The femoral artery, after giving off the profunda, which was somewhat larger than common, continued its course below the sartorius, supplying branches to the muscles in its neighbourhood, particularly towards the middle of the thigh, where a greater number of small vessels than usual were sent off from it. It then terminated abruptly, and was obliterated for the space of one inch, where the ligature had been applied in the operation. Below this it received large branches on each side from the vessels coming from the profunda, and passed, as usual, through the tendon of the triceps into the ham. The diameter of the artery was but little diminished, even at the very point of its termination, where it formed a complete cul-de-sac; and there was no perceptible difference in its size where it again admitted the injection. A very free communication took place between the popliteal artery and the branches of the profunda, and also between these and the artery below the knee. One of the anastomosing branches, formed by vessels coming from the profunda uniting with others from the poplitea, became a trunk as large as the upper part of the ulnar artery. It run down, deep-seated between the outer and inner ham-strings, immediately behind the poplitea, and divided into two branches, one passing over each of the condyles of the femur to join the principal artery below the remains of the aneurismal sac. The popliteal artery, on entering the notch between the condyles of the femur, sent off a very large branch towards the outside of the knee, joining the anastomosing vessels there, and immediately afterwards became obliterated for the space of two and a half inches, where the aneurismal sac had been situated. This space was supplied by a dark-coloured ligamentous substance, which united the two ends of the vessel. The same substance occupied the place where the artery was obliterated in the middle of the thigh. On again becoming pervious, the artery, for the space of an inch, was not larger than the tibialis antica. It then received the anastomosing vessels from the outer and inner condyles, and divided, as usual, into the two tibial and the peroneal arteries.

